# South-West Orthopaedic Club

**Published:** 1973-07

**Authors:** 


### South-West Orthopaedic Club

A Meeting of the Club with members of the Irish Orthopaedic Association and the Hellenic Society of Orthopaedic Surgery and Traumatology was held at the Royal Cornwall Hospital, Truro, on the 19th May, 1973.

**Fibromatous Lesions in Orthopaedics**—Dr. G. Stewart Smith (Exeter).

The clinical histories and histopathological features of a series of fibromatous lesions in orthopaedic cases were reviewed including Dupuytren's contracture, plantar fasciitis, juvenile aponeurotic fibroma, desmoid, pseudosarcomatous fasciitis and various patterns of musculo-aponeurotic fibromatosis.

Special attention was given to conditions of musculo-aponeurotic fibromatosis, some of which on histological grounds had originally been diagnosed as fibrosarcoma. Although these lesions recurred sometimes more than once and infiltrated widely they did not metastasise and were considered to be non-malignant. D. H. Mackenzie's view that the term fibrosarcoma should be reserved for tumours with the power to metastasise was supported and his conception of a "third world of fibroblastic behaviour" accepted.

**The Late Results of Osteotomy in Osteoarthritis of the Hip**—Dr. Dretakis (Athens) reported on the results obtained in 112 hips which he had been able to follow up for a minimum of 5 years. The better results had been obtained in patients with primary osteoarthritis and in those with the least severe changes. Better results had been obtained where internal fixation had been used in preference to external fixation. In relation to the amount of displacement produced at the osteotomy he had found that where no displacement was introduced, pain relief had initially been good but had not lasted. A displacement of more than half the diameter often resulted in slow union. In the whole series 20 osteotomies had been slow to unite and 2 had gone on to non-union. Siting the osteotomy below the level of the lesser trochanter also appeared to have an adverse effect upon the union rate.

**Deformities of the Ankle due to Epiphyseal Injury**—Mr. J. D. Wrighton (Exeter) first discussed the general classification of epiphyseal injuries. In the ankle region epiphyseal damage more commonly resulted in a varus deformity. Less commonly a valgus deformity might be produced and such a case was demonstrated.

**The Development of Metal Sensitivity as a Complication of Total Hip Replacement**—Mr. E. Mervyn Evans (Swansea).

Three cases were described in which it was suggested that sensitivity to one of the components of a McKee Farrar prosthesis had led to fragmentation of the acetabulum and loosening of the prosthesis. L.H. developed progressive fragmentation in both hips 18 months after replacement. There was no evidence of infection but skin tests revealed a strong sensitivity to cobalt with negative results for chromium, nickel and cement. Both prostheses were removed and the joints were sterile but contained grey pultaceous material with a relatively high cobalt and chromium content; there were multiple acetabular fractures on each side and the acetabular wall was paper-thin. Following the removal of the second prosthesis she became cobalt-negative and her urinary cobalt which had been raised returned to normal.

N.R. developed similar symptoms 18 months after replacement. She was cobalt sensitive and her X-ray and operative findings were the same as in the first case reported.

M.W. presented as a case of femoral loosening 2 years after replacement. She was found to be chromium sensitive and again the joint contained grey material with large quantities of cobalt and chromium. 30 cases of 2 years old prosthetic replacement cases without symptoms and 30 controls have been tested for skin sensitivity to metals and all have proved negative.

**Ankylosing Spondylitis—an Optimistic View of Management**—Gp. Capt. Wynn Parry had reviewed a total of 242 cases of ankylosing spondylitis. He drew attention to the importance of diagnosing the disease before X-ray changes appeared in the sacro-iliac joints and the E.S.R. became elevated. A history of low back pain relieved by exercise and made worse by rest together with limitation of lateral lumbar flexion as well as forward flexion and a restriction of chest expansion below 5 cm. strongly suggests a diagnosis of this disease. With a regime of exercise, drug therapy and periodic admission to a Rehabilitation Centre for intensive mobilisation, the prognosis was much better than usually believed — 63% of the patients he had studied had been able to follow a full Service career. Phenylbutazone in doses up to 600 mg a day had been found to be the most valuable drug in the early stages of treatment in relieving pain and stiffness. Once maximum mobility had been achieved it was often possible to reduce the dosage or even stop the drug altogether, resuming only if there were a relapse. Exercise was considered to be very important and all patients should be strongly encouraged to take up regular sports, avoiding only violent contact sports. By following such a regime the prognosis for this disease had proved to be good and the view was now taken that all patients should be retained in the Service. Of 66 patients treated in the last 9 years, 62 were still on full duty. Some improvement in the range of spinal movement and chest expansion had been obtained in a high proportion, and it had been possible to prevent the deformities once so common in this disease.

**Flexor Tendon Grafting** (film)—Dr. R. Tubiana (Paris).

